# Supplementary material for: G-CSF/NAMPT signaling drives neutrophil dysfunction and enhances bacterial infection susceptibility in cancer patients
Source: Nat Commun. 2025 Dec 12;16:11137. doi: 10.1038/s41467-025-67471-4 (PMC12705696; doi:10.1038/s41467-025-67471-4)
Supplement: Supplementary file 1 — Supplementary Information [file 41467_2025_67471_MOESM1_ESM.pdf]

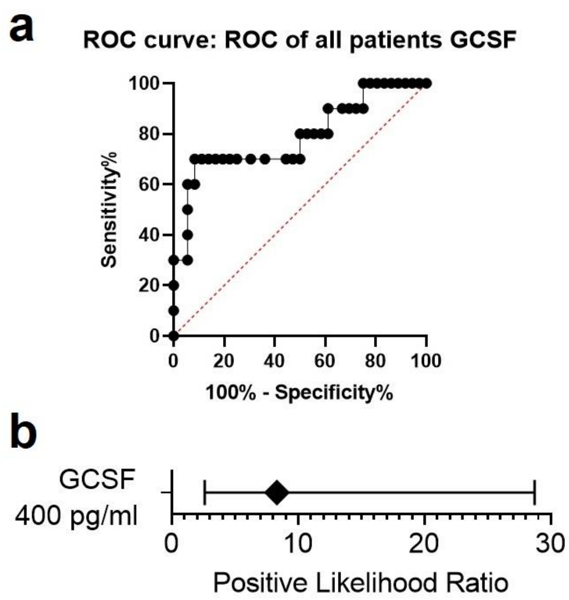

Figure S1 | Association between G-CSF levels in oral rinse and presence of Gram-negative pathogens.

(a) Receiver operating characteristic (ROC) curve assessing the ability of G-CSF concentration in oral rinse to predict the presence of Gram-negative pathogens in the oral cavity across all participants (n=46). A concentration > 400 pg/ml was identified as the optimal threshold for prediction.
(b) Relative risk of detecting Gram-negative pathogens in participants with oral rinse G-CSF concentrations > 400 pg/ml, showing an 8.4-fold higher risk compared with those below the threshold.

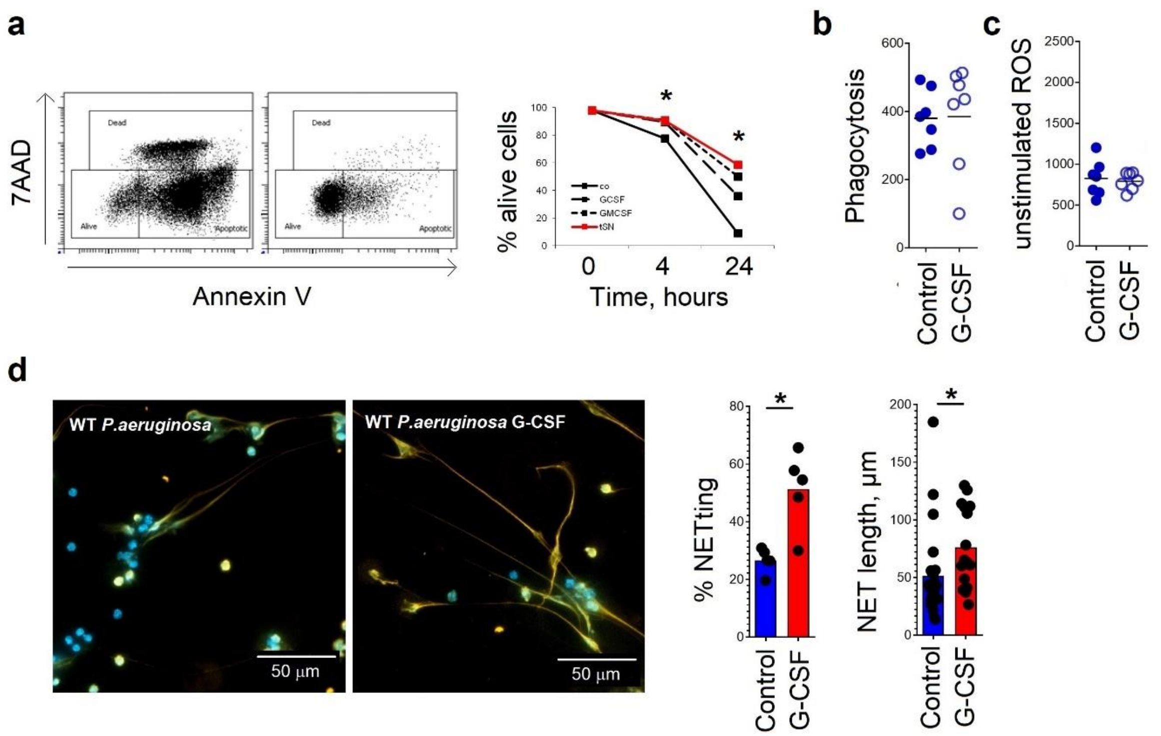

Figure S2 | Granulocyte colony-stimulating factor (G-CSF) modulates neutrophil survival and effector functions.

(a) Flow cytometry plots (Annexin V/7AAD staining) show viability of neutrophils under different conditions (left), with quantification (right) depicting the percentage of alive cells over time in control (co), G-CSF, GM-CSF, and tSN (tumor supernatant) conditions. Both G-CSF and tSN significantly delay neutrophil apoptosis compared to control at 4 and 24 h.
(b) Phagocytosis of *Pseudomonas aeruginosa* by neutrophils is shown (n=7 biological replicates), with G-CSF treatment compared to control.
(c) Unstimulated reactive oxygen species (ROS) production is quantified in control versus G-CSF-treated neutrophils (n=7 biological replicates).
(d) Representative fluorescence images (left) and quantification (right) of neutrophil extracellular trap (NET) formation and NET length following stimulation with *P. aeruginosa* in the presence or absence of G-CSF. G-CSF significantly increases both the percentage of NET-forming cells and NET length (n=5 biological replicates).
G-CSF, granulocyte colony-stimulating factor; GM-CSF, granulocyte-macrophage colony-stimulating factor; tSN, tumor supernatant; ROS, reactive oxygen species; NET, neutrophil extracellular trap; 7AAD, 7-aminoactinomycin D; co, control. Data are presented as means with individual values shown. Scale bars: 50 µm (d, fluorescence images). Statistical significance was determined using unpaired two-tailed t-test or one-way ANOVA with multiple comparisons, as appropriate.
$*p < 0.05$.

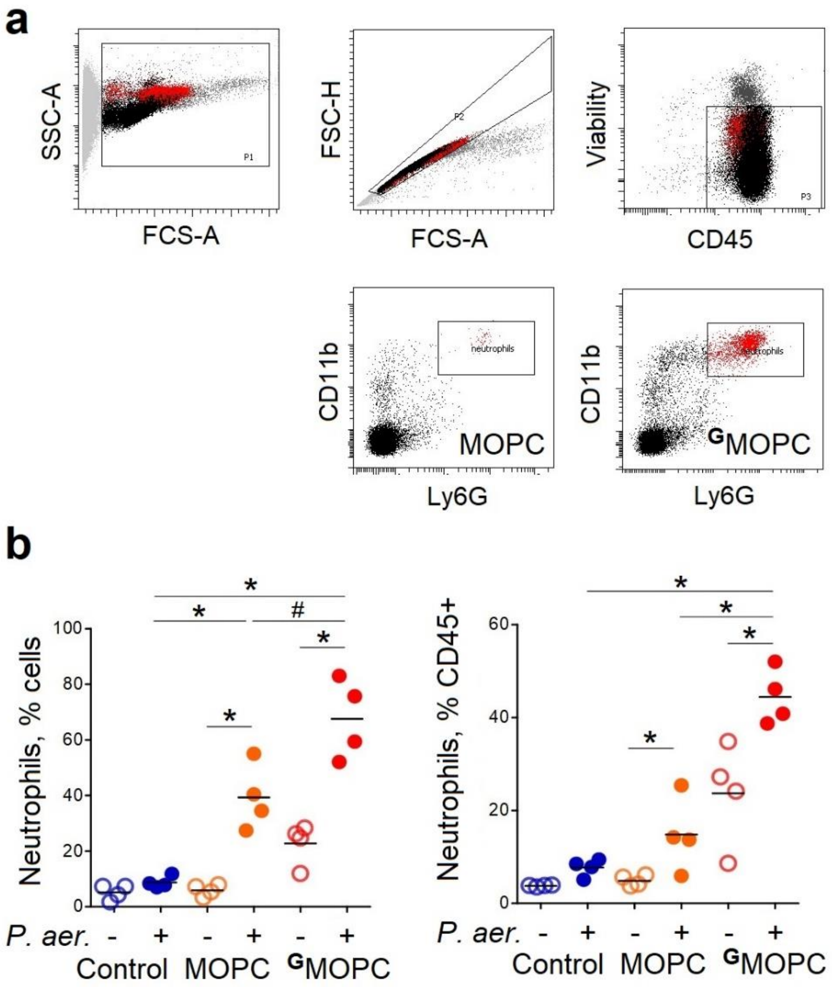

Figure S3 | Flow cytometric analysis of neutrophil populations in lung tissue following infection.

(a) Representative flow cytometry gating strategy for identification of neutrophils in lung tissue. Cells were gated on forward and side scatter (FCS-A vs. SSC-A, left panel, gate P1), followed by doublet exclusion (FCS-A vs. FCS-H, middle panel, gate P2). Dead cells were excluded by viability dye and CD45+ leukocytes were selected (right panel, gate P3). Neutrophils were further defined as CD11b+Ly6G+ cells in mice treated with either MOPC (middle bottom panel) or ^{G}MOPC (right bottom panel); neutrophils are indicated in red.

(b) Quantification of neutrophil frequencies in lung tissue following *P. aeruginosa* infection. The percentage of neutrophils among total cells (left) and among CD45+ leukocytes (right) are shown for control (no tumor), MOPC, and ^{G}MOPC groups, with or without *P. aeruginosa* infection (n=4 biological replicates). Each symbol represents an individual mouse; horizontal bars indicate mean values. * indicates $p < 0.05$, # indicates $p = 0.06$.

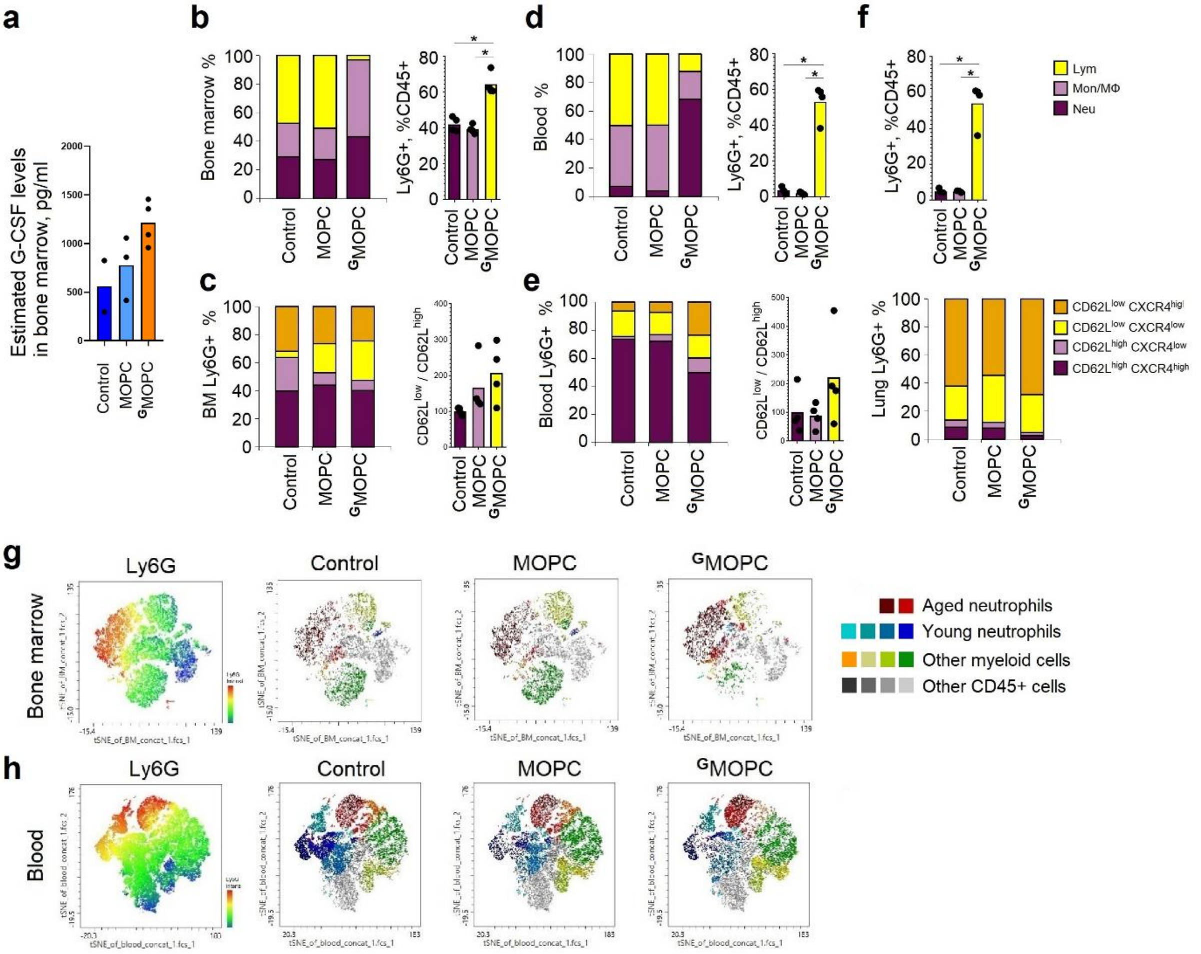

Figure S4 | Characterization of neutrophil populations in lung tissue, bone marrow, and blood in control, MOPC, and ^{G}MOPC-bearing mice.

(a) Estimated granulocyte colony-stimulating factor (G-CSF) concentrations in bone marrow of tumor-free (n=2) MOPC- (n=3) and ^{G}MOPC-bearing mice (n=4), measured by ELISA (mean ± s.e.m.; *P < 0.05, Mann-Whitney test).

(b–f) Flow cytometric quantification and phenotyping of leukocyte populations. Stacked bar plots show proportions of major leukocyte populations (lymphocytes [Lym], monocytes/macrophages [Mon/Mø], neutrophils [Neu]) and neutrophil subsets (CD62L^{low} CXCR4^{high}, CD62L^{low} CXCR4^{low}, CD62L^{high} CXCR4^{low} , CD62L^{high} CXCR4^{high}) in bone marrow (b,c respectively), blood (d,e respectively), and lung tissue (f) from control, MOPC, and ^{G}MOPC-bearing mice (n=4 biological replicates in each group). Dots represent individual animals; horizontal lines indicate mean values; *P < 0.05 by Mann-Whitney test.
(g, h) t-SNE plots of neutrophil populations in bone marrow (g) and blood (h) from control, MOPC, and ^{G}MOPC-bearing mice, colored by Ly6G expression (left panels) and indicating clustering of Ly6G+CD62L^{low}CD11b^{low} neutrophils ("aged", highlighted in red) and Ly6G+CD62L^{high}CD11b^{high} ("young", highlighted in red). t-SNE axes are labeled with analysis parameter numbers; color scales indicate expression intensity.

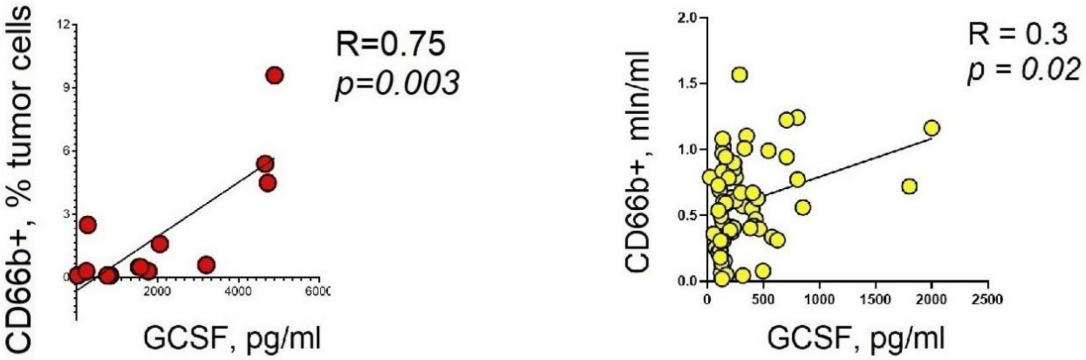

Figure S5 | Correlation between G-CSF levels and neutrophil (CD66b+) abundance in human tumor tissue and oral rinse samples.

(a) Scatter plot showing the relationship between G-CSF concentration and the percentage of CD66b+ neutrophils among tumor cells isolated from human lung tumor tissue. Each dot represents an individual patient sample (n=12). Spearman's correlation analysis revealed a strong positive association (R=0.75, p=0.003).
(b) Scatter plot depicting the correlation between G-CSF levels (pg/ml) and absolute CD66b^+ neutrophil counts (mln/ml) in matched human oral rinse samples. Each dot represents a single sample (n=61). Spearman's correlation analysis demonstrates a moderate positive association (R=0.3, p=0.02).

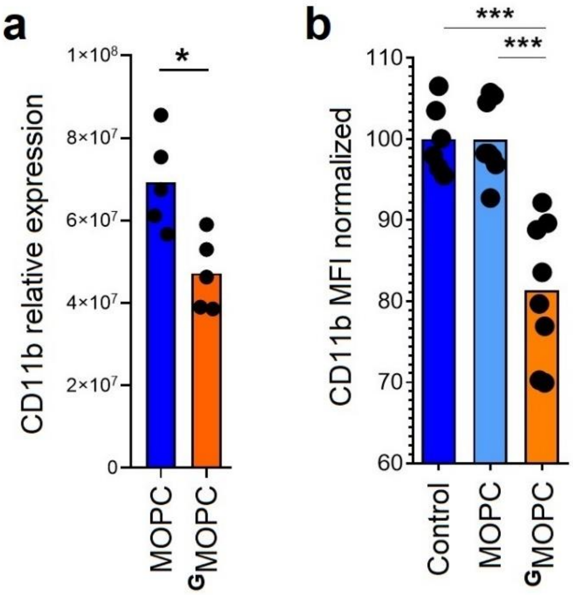

Figure S6 | Quantification of CD11b expression in lung neutrophils by proteomics and flow cytometry in control, MOPC, and ^{G}MOPC-bearing groups.

(a) Bar graph shows relative CD11b expression in lung neutrophils measured by proteomics analysis from MOPC-treated (blue) and ^{G}MOPC-treated (orange) mice (n=5 in each group). Each dot represents an individual animal; bars indicate mean values. Statistical significance was determined by Mann-Whitney test (*P < 0.05).
(b) CD11b median fluorescence intensity (MFI), normalized to the control group, in lung neutrophils assessed by flow cytometry in control (n=6), MOPC (n=8), and ^{G}MOPC-bearing mice (n=8). Each dot represents an individual sample; bars indicate mean values. Statistical analysis by Mann-Whitney test; ***P < 0.001.
Horizontal lines indicate group means.

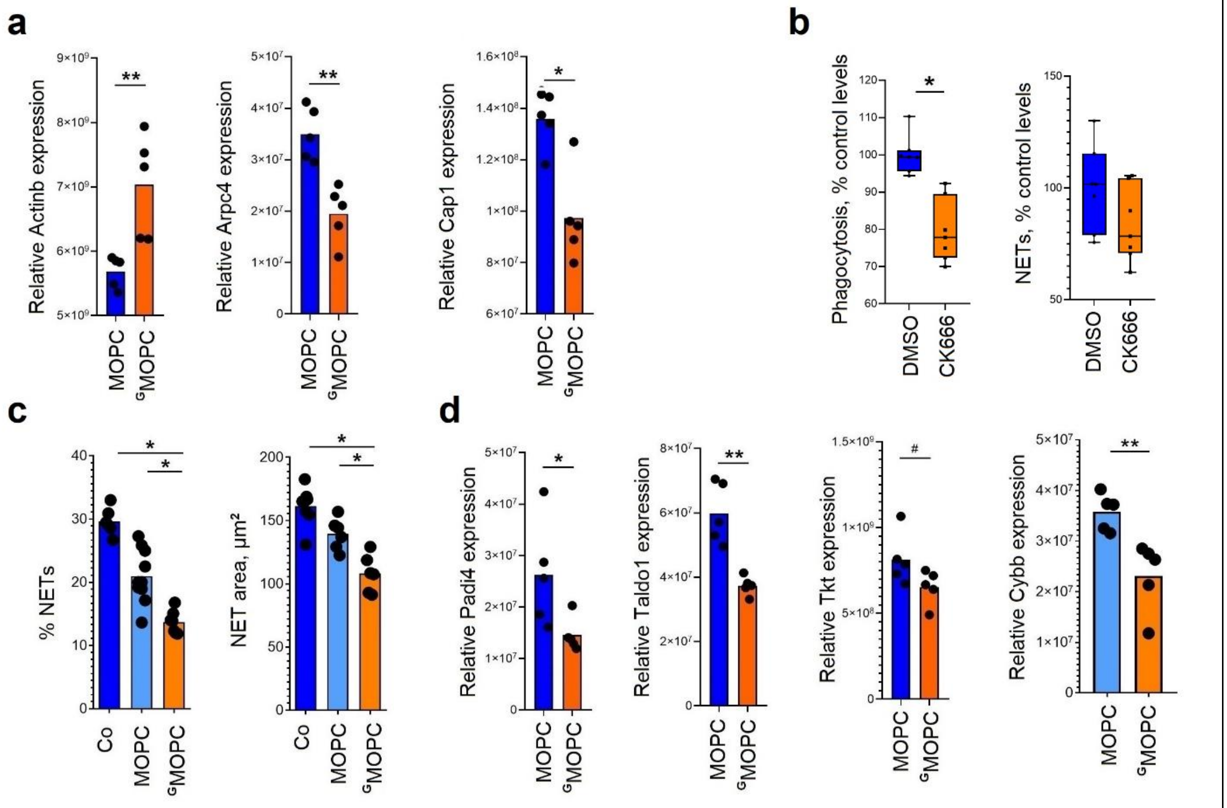

Figure S7 | Regulation of actin branching and NET formation in lung neutrophils from MOPC- and ^{G}MOPC-beating mice.

(a) Quantitative proteomics analysis of proteins involved in actin branching in lung neutrophils, comparing MOPC-treated (blue) and ^{G}MOPC-bearing (orange) groups. Relative expression levels of Actb (β-actin), Arpc4 (Arp2/3 complex subunit), and Cap1 (adenylate cyclase-associated protein 1) are shown. Each dot represents an individual animal (n=5 in each group); bars indicate mean values. Statistical significance is shown (*P < 0.05, **P < 0.01, Mann-Whitney test).
(b) *In vitro* inhibition of actin branching with CK666 decreases phagocytosis in neutrophils, compared to vehicle (DMSO) controls (left panel), as measured by percent control levels. Right panel: trend to decrease of NET by CK666 treatment. N=7 biological replicates in each group, box plots show median and interquartile ranges; *P < 0.05, Mann-Whitney test.
(c) NET formation by lung neutrophils from control (Co, n=6), MOPC (n=10), and ^{G}MOPC (n=7) groups as assessed by percentage of NET-positive cells (left) and NET area per cell (µm², right). Each dot denotes an individual animal; bars depict mean values. Statistical analysis by Mann-Whitney test; *P < 0.05.
(d) Proteomics evaluation of proteins implicated in NETosis in lung neutrophils, comparing MOPC and ^{G}MOPC groups (n=5 mice in each group). Relative expression levels of Padi4 (peptidyl arginine deiminase 4), Taldo1 (transaldolase 1), Tkt (transketolase), and Cybb (cytochrome b-245 beta chain) are presented. Dots indicate individual samples; bars show mean values. Statistical significance is indicated (*P < 0.05, **P < 0.01, #P < 0.1, Mann-Whitney test).
Horizontal lines represent mean group values.

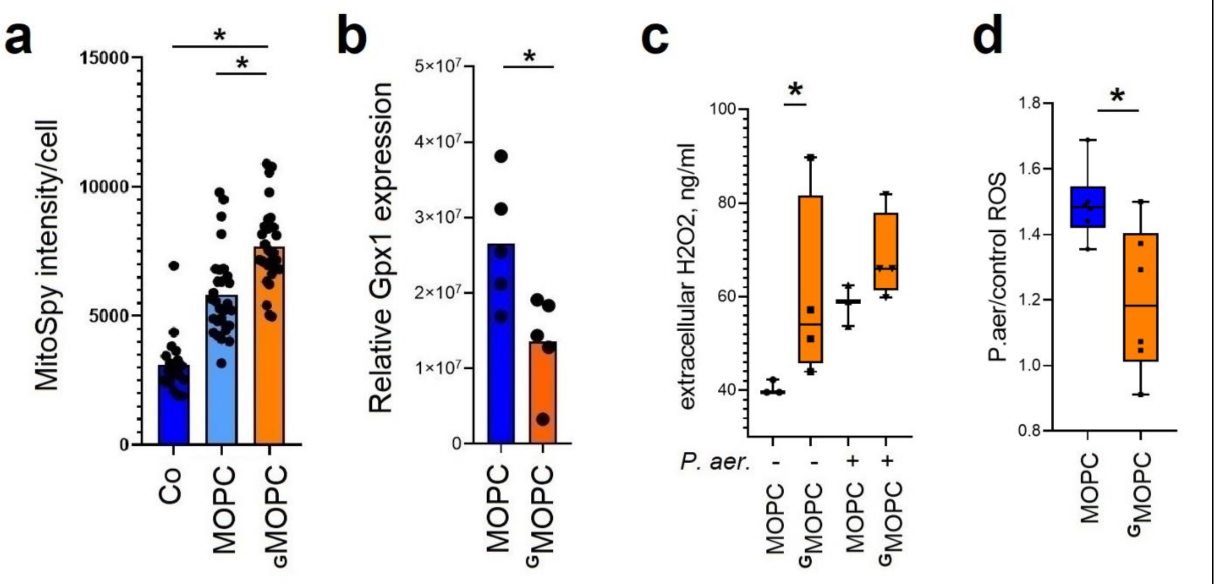

Figure S8 | Altered mitochondrial function and ROS activity in lung neutrophils from MOPC- and ^{G}MOPC-bearing mice.

(a) Quantification of mitochondrial mass in lung neutrophils using MitoSpy fluorescence intensity (arbitrary units per cell). Neutrophils from control (Co, n=20), MOPC-treated (n=30), and ^{G}MOPC-bearing (n=30) mice (n=4 in each groups, pooled) were analyzed by flow cytometry; each dot represents an individual mouse. Bars indicate mean values; statistical significance by Mann-Whitney test (*p < 0.05).

(b) Relative expression of Gpx1 (glutathione peroxidase 1) mRNA in lung neutrophils from MOPC- and ^{G}MOPC-bearing mice (n=5 mice in each group), determined by quantitative proteomics. Dots indicate individual biological replicates; bars show mean values. Statistical significance by Mann-Whitney test (*p < 0.05).

(c) Extracellular hydrogen peroxide (H2O2 ng/ml) released by lung neutrophils from MOPC (n=3 mice) and ^{G}MOPC-bearing mice (n=4 mice), with (+) or without (–) *Pseudomonas aeruginosa* stimulation. Data are presented as box-and-whisker plots; *p < 0.05 by Mann-Whitney test.

(d) Ratio of reactive oxygen species (ROS) generation upon *P. aeruginosa* challenge relative to unstimulated control in lung neutrophils from MOPC and ^{G}MOPC-bearing mice (n=6 in each group). Box-and-whisker plots depict interquartile ranges and medians; statistical significance by Mann-Whitney test (*p < 0.05).

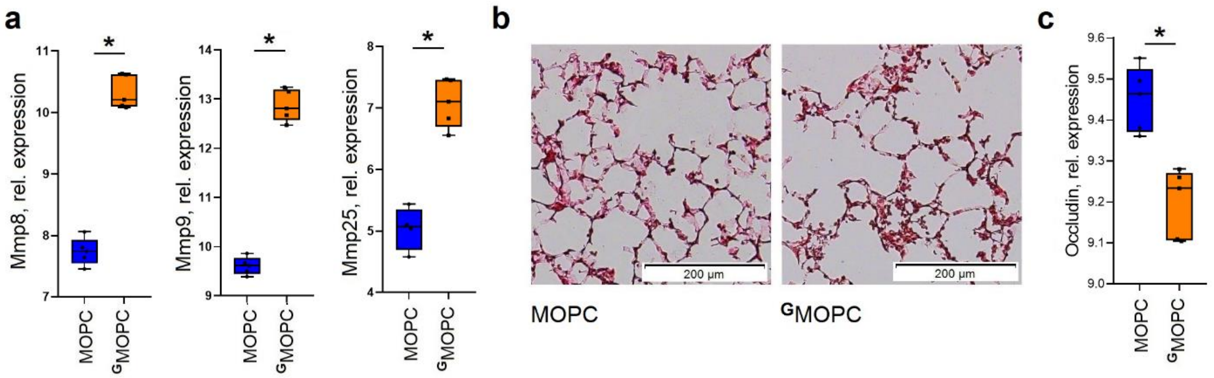

Figure S9 | ECM remodeling and barrier integrity alterations in lungs from MOPC- and ^{G}MOPC-bearing mice.

(a) Relative protein expression of matrix metalloproteinases (Mmp8, Mmp9, and Mmp25) in lung tissue, analyzed by quantitative proteomics (n=5 mice in each group). Box-and-whisker plots show interquartile ranges and medians for MOPC (blue) and ^{G}MOPC-bearing (orange) groups; $^{*}P < 0.05$, Mann–Whitney U test.
(b) Representative images of lung sections stained with Van Gieson for collagen detection in MOPC and ^{G}MOPC-bearing mice. Collagen fibers appear red; scale bar = 200µm.
(c) Relative expression levels of occludin protein in lung tissue, assessed by proteomics (n=5 mice in each group). Box plots indicate median and interquartile ranges for MOPC and ^{G}MOPC-bearing groups; $^{*}P < 0.05$, Mann–Whitney U test.

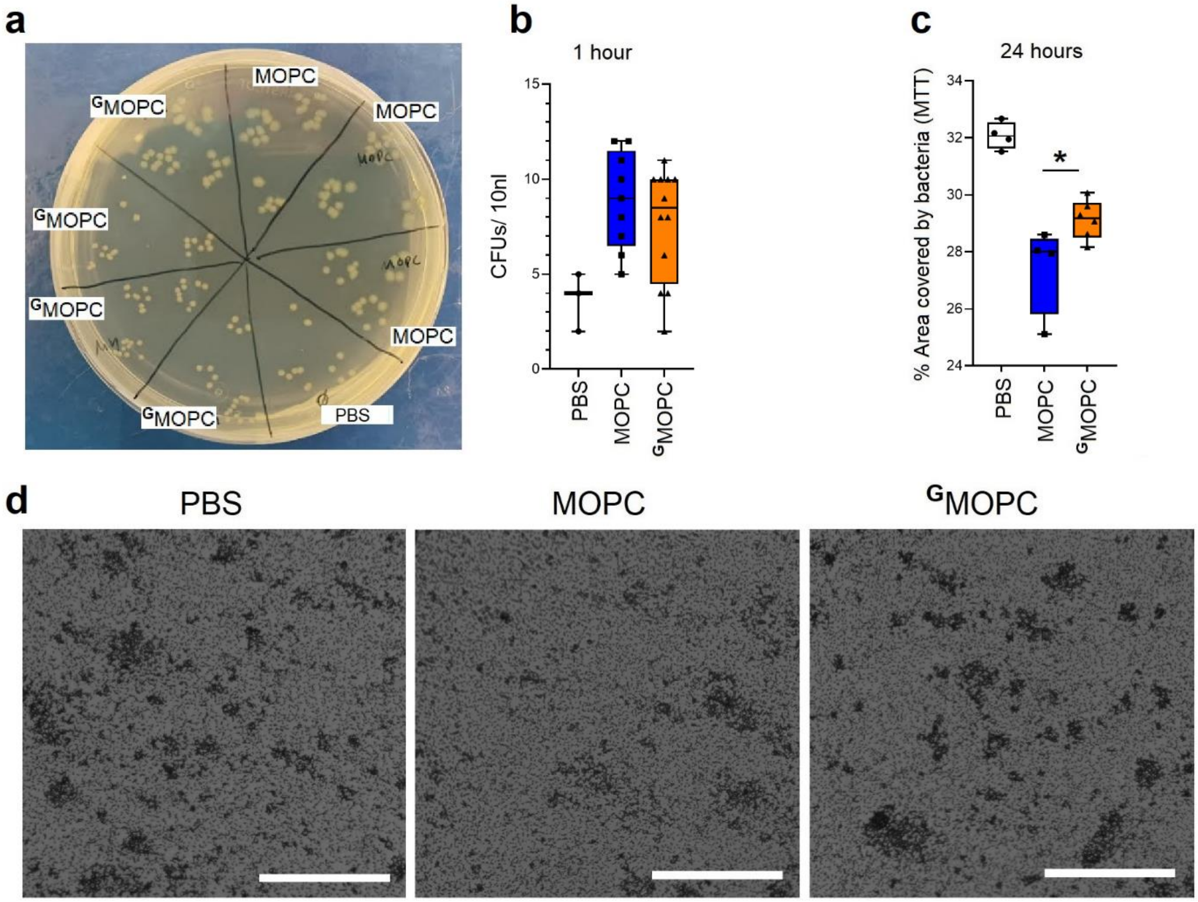

Figure S10 | Antibacterial activity of lung neutrophils from MOPC- and ^{G}MOPC-bearing mice against *Pseudomonas aeruginosa*.

(a) Representative image of an agar plate showing colony-forming units (CFUs) of *P. aeruginosa* following co-culture with PBS or lung neutrophils isolated from mice bearing MOPC, ^{G}MOPC. Plates are segmented by treatment condition.
(b) Quantification of *P. aeruginosa* CFUs recovered after 1 hour of co-culture with PBS (n=3) or lung neutrophils from each experimental group (MOPC n=9, ^{G}MOPC n=12 technical replicates). Box plots display median and interquartile range. No significant differences between MOPC, ^{G}MOPC groups (Mann-Whitney test).
(c) Quantification of bacterial spread (% area covered by bacteria, determined by MTT assay) after 24 hours of co-culture with PBS (n=4 mice) or neutrophils from MOPC (n=4 mice) and ^{G}MOPC (n=5 mice). Box plots indicate median and interquartile range; *$p < 0.05$ by Mann-Whitney test.
(d) Representative images of the plate bottom after 24-hour co-culture, stained with crystal violet to visualize remaining bacteria. Scale bars, 200 μm.

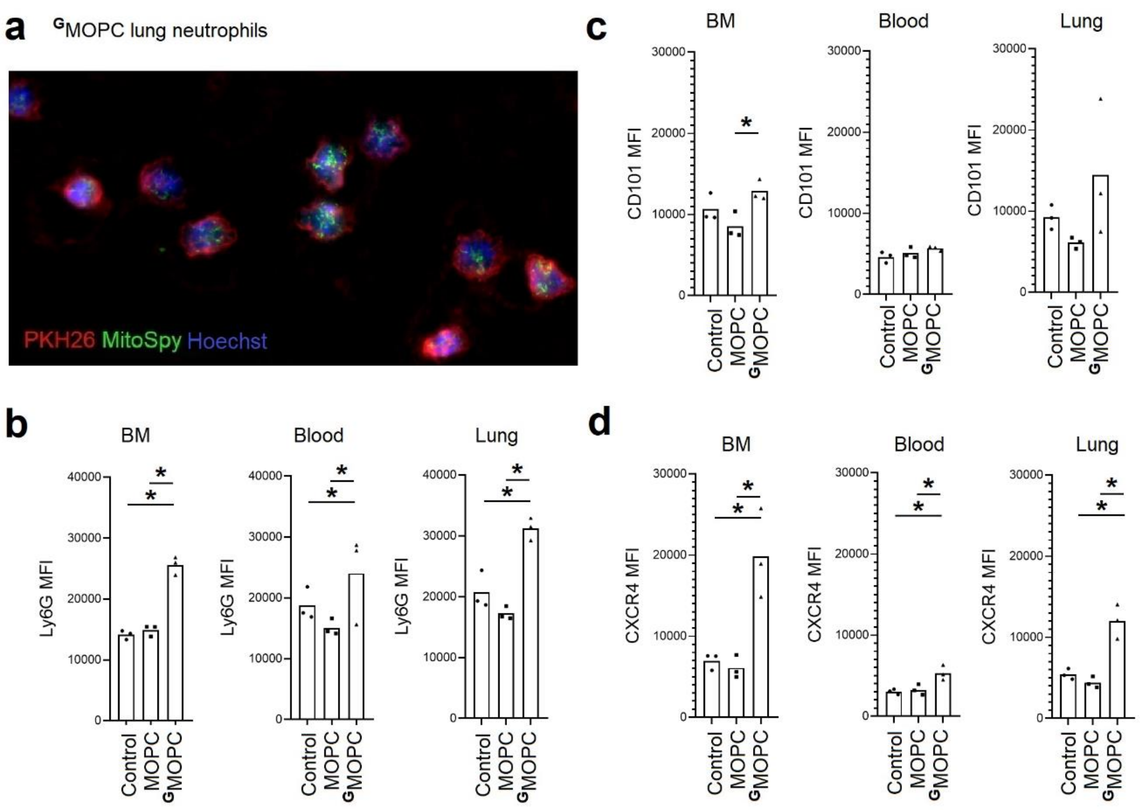

Figure S11 | Phenotypic characterization of ^{G} neutrophils.

(a) Representative fluorescence microscopy image of ^{G}neutrophils isolated from lungs showing segmented nuclei stained with Hoechst (blue), cytoplasm labeled with PKH26 (red), and mitochondria with MitoSpy (green).
b-d. Quantification of Ly6G (b), CD101 (c), CXCR4 (d) expression (median fluorescence intensity, MFI) in neutrophils from bone marrow (BM), blood, and lung tissue in control tumor-free (n=3), MOPC- (n=3), and ^{G}MOPC-bearing (n=3) mice, assessed by flow cytometry. Each bar represents median and interquartile range; $*p < 0.05$, Mann-Whitney test.

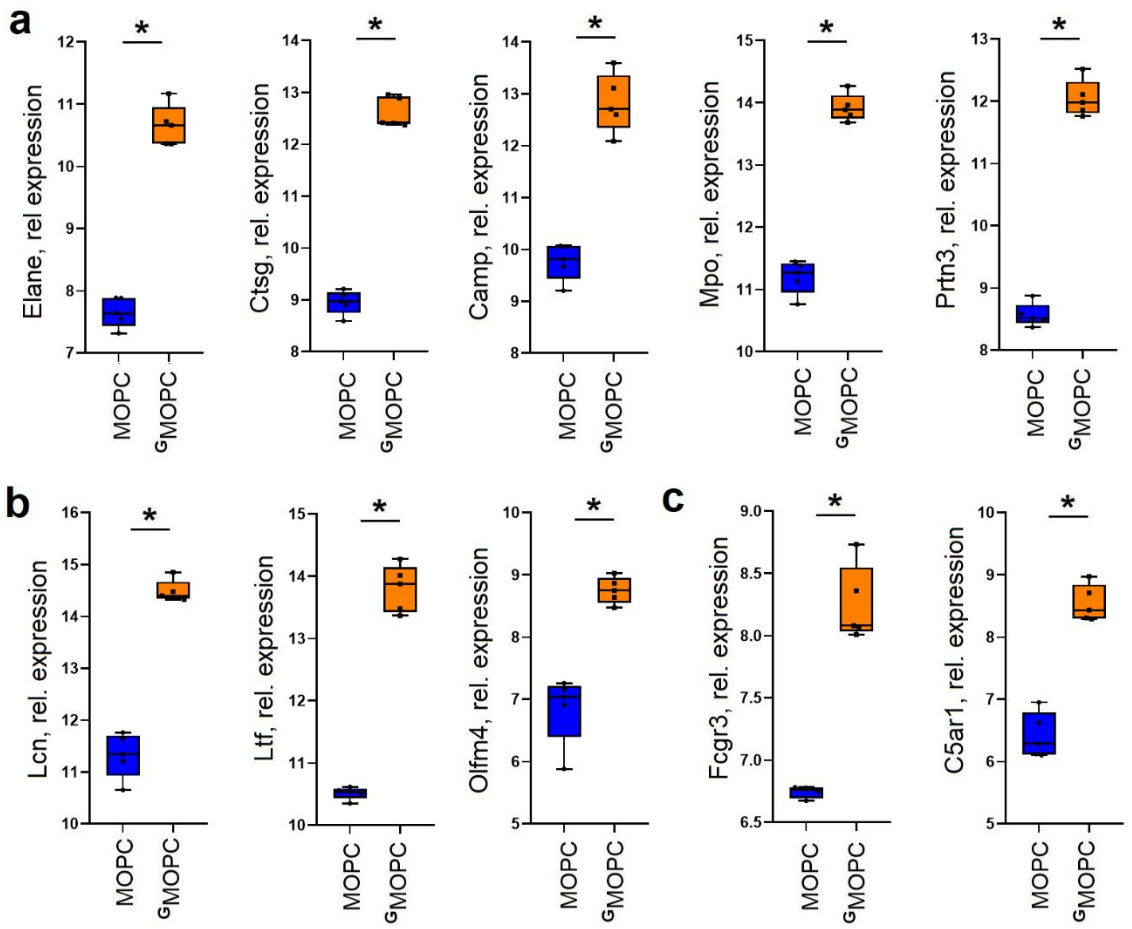

Figure S12 | Differential expression of neutrophil granule proteins in lung tissue from MOPC- and GMOPC-bearing mice, assessed by proteomics.

(a) Relative expression levels of proteins associated with primary (azurophilic) granules in lung tissue. Box-and-whisker plots show expression of Elane (neutrophil elastase), Ctsg (cathepsin G), Camp (cathelicidin antimicrobial peptide), Mpo (myeloperoxidase), and Prtn3 (proteinase 3) in neutrophils from MOPC (blue) and GMOPC (orange) groups (n=5 in each group). *$P < 0.05$ by Mann–Whitney U test.
(b) Quantitative proteomics of secondary (specific) granule proteins. Shown are Lcn (lipocalin 2), Ltf (lactotransferrin), and Olfm4 (olfactomedin 4) in neutrophils from MOPC (blue) and GMOPC (orange) groups (n=5 in each group); box-and-whisker plots indicate median and interquartile range. *$p < 0.05$ by Mann–Whitney U test.
(c) Analysis of secretory vesicle proteins FcgR3 (CD16) and C5ar1 (C5a receptor 1) in lung neutrophils from MOPC (blue) and GMOPC (orange) groups (n=5 in each group). Relative expression is presented as box-and-whisker plots; *$p < 0.05$ by Mann–Whitney U test.

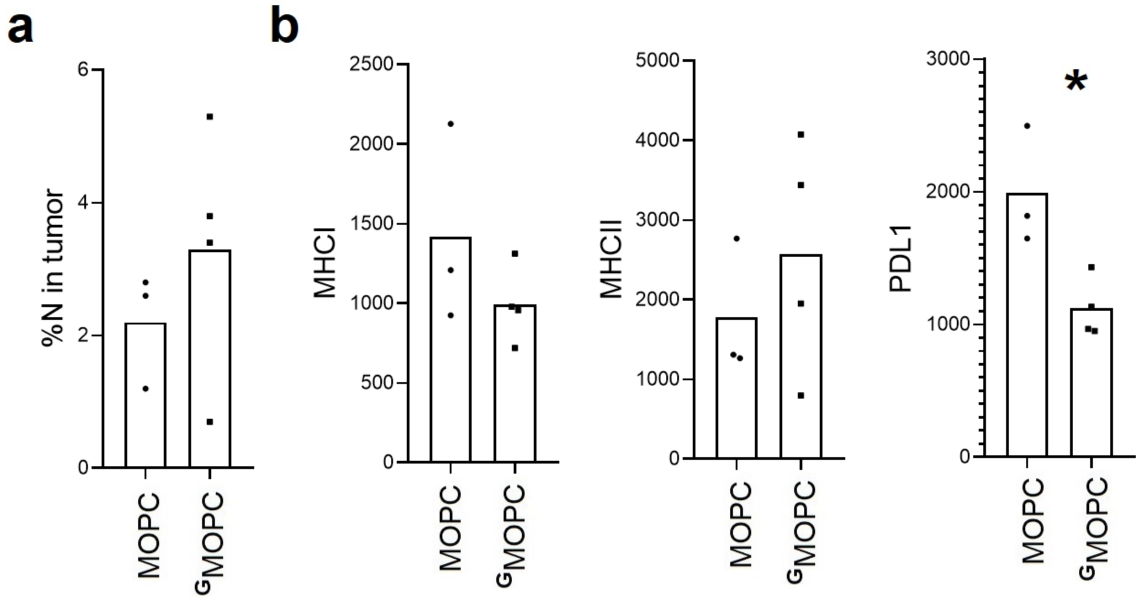

Figure S13 | Frequency and immunoregulatory marker expression in lung neutrophils from MOPC- and ^{G}MOPC-bearing mice.

(a) Percentage of neutrophils (%N) among total cells in tumor tissue from MOPC (n=3 mice) and ^{G}MOPC (n=3 mice) groups, determined by flow cytometry. Bars represent mean ± s.e.m. per group, Mann-Whitney test.
(b) Expression levels of immunoregulatory markers in tumor neutrophils: surface major histocompatibility complex class I (MHCI, left), class II (MHCII, middle), and programmed death-ligand 1 (PDL1, right), as quantified by median fluorescence intensity (MFI). Values are shown for MOPC (n=3 mice) and ^{G}MOPC (n=3 mice) groups. Bars indicate mean ± s.e.m.; *P < 0.05 by Mann-Whitney test.

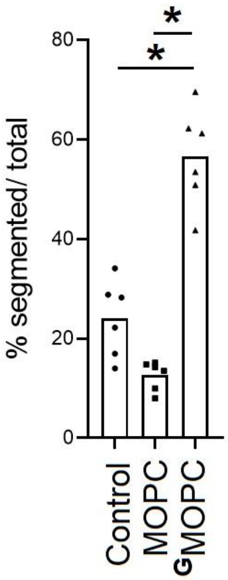

Figure S14 | Maturation status of BM progenitors *in vitro*.

Bone marrow progenitor cells isolated from n=6 tumor-naïve mice were cultured in the presence of MOPC, ^{G}MOPC-conditioned medium or control medium for 6 days, then cytospins were prepared. Calculation of cells with segmented nucleus, % from total cells in a fiels of view (Mann-Whitney test, *p < 0.05).

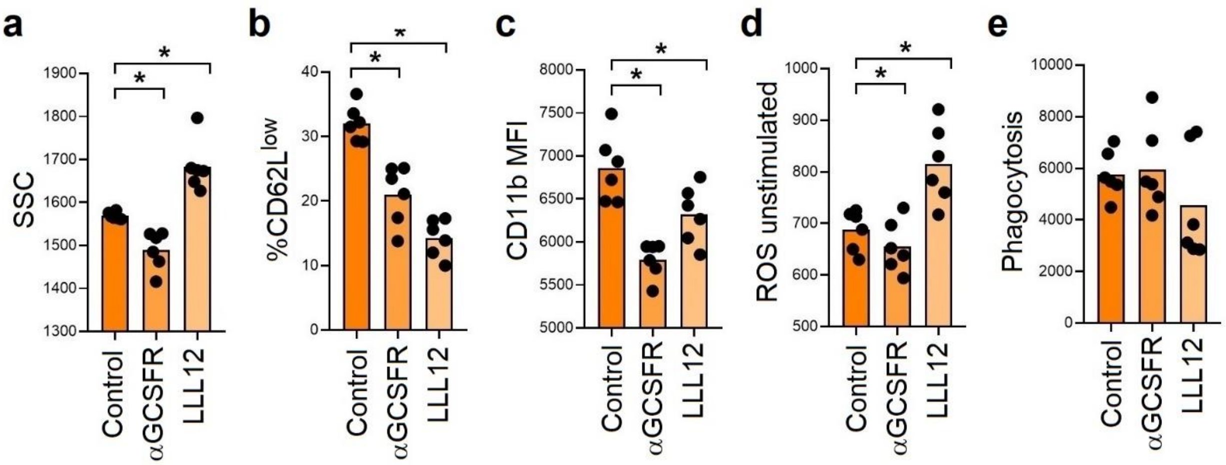

Figure S15 | *In vitro* maturation of bone marrow progenitors in ^{G}MOPC-conditioned medium with G-CSF signaling inhibition.

(a–e) Bone marrow progenitor cells isolated from n=6 tumor-naïve mice were cultured in the presence of ^{G}MOPC-conditioned medium and treated with G-CSF signaling inhibitors (αGCSFR antibody or LLL12) or control medium. Quantification includes: (a) side scatter (SSC), (b) percentage of CD62L^{low} neutrophil subset, (c) CD11b median fluorescence intensity (MFI), (d) reactive oxygen species (ROS) levels in unstimulated neutrophils, and (e) phagocytic activity. Each dot represents an independent replicate; bars show mean values. Asterisks indicate statistically significant differences between groups (Mann-Whitney test, *P < 0.05)

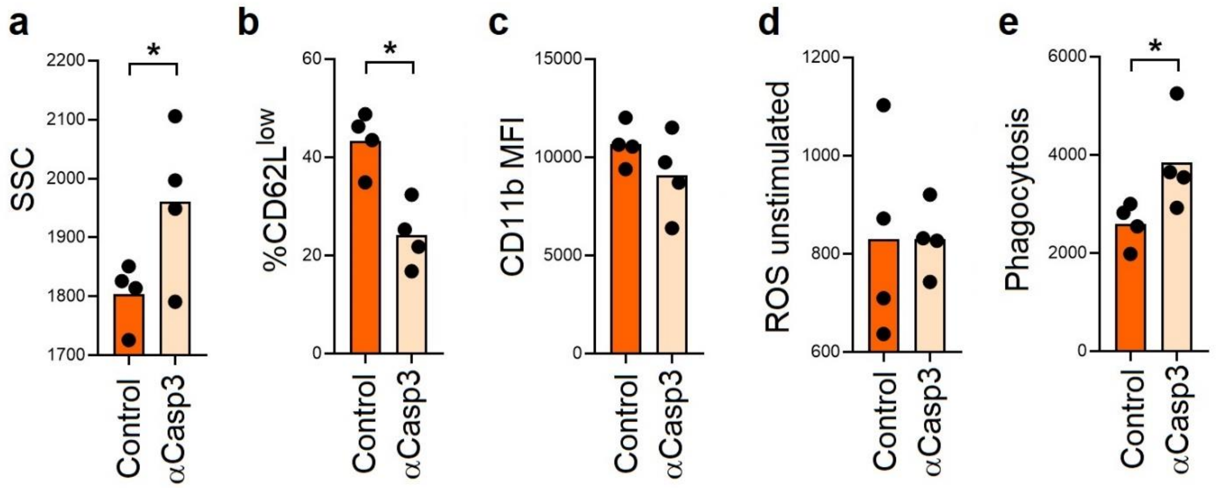

Figure S16 | *In vitro* maturation of bone marrow progenitors in ^{G}MOPC-conditioned medium with Caspase 3 inhibition.

(a–e) Bone marrow progenitor cells isolated from n=4 tumor-naïve mice were cultured in the presence of ^{G}MOPC-conditioned medium and treated with Caspase 3 inhibitor QVD-OPh or control medium. Quantification includes: (a) side scatter (SSC), (b) percentage of CD62L^{low} neutrophil subset, (c) CD11b median fluorescence intensity (MFI), (d) reactive oxygen species (ROS) levels in unstimulated neutrophils, and (e) phagocytic activity. Each dot represents an independent replicate; bars show mean values. Asterisks indicate statistically significant differences between groups (Mann-Whitney test, *$P < 0.05$)

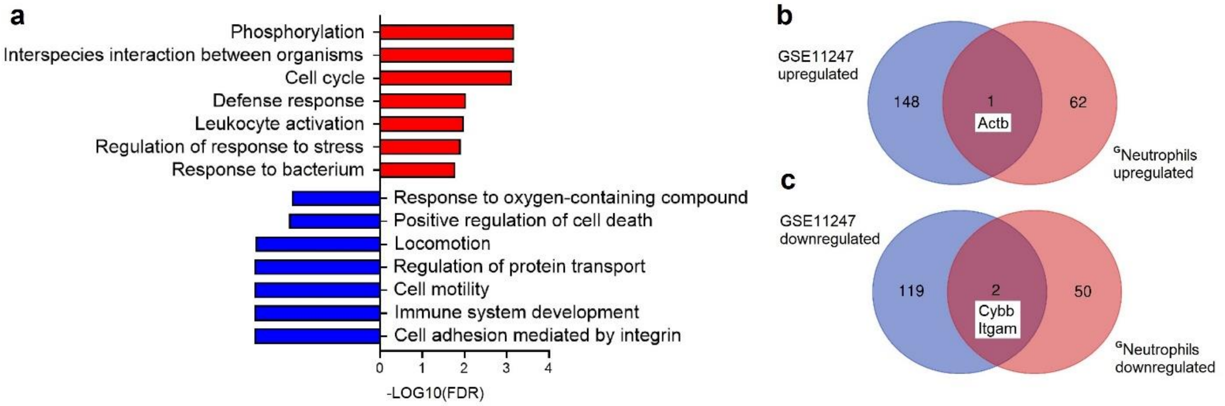

Figure S17 | Pathway analysis of differentially regulated processes in G-CSF-induced CD133+ stem cells and overlap with ^{G}neutrophils.

(a) Gene ontology (GO) pathway enrichment analysis of genes differentially expressed in G-CSF-induced stem cells. Bar graphs display pathways significantly upregulated (red) and downregulated (blue), based on –log10(FDR). Upregulated pathways include phosphorylation, interspecies interaction, cell cycle, defense response, leukocyte activation, regulation of response to stress, and response to bacterium. Downregulated pathways involve response to oxygen-containing compounds, cell death regulation, locomotion, protein transport, cell motility, immune system development, and cell adhesion mediated by integrin.

(b, c) Venn diagrams showing overlap between differentially expressed genes in G-CSF-induced stem cells (GSE11247) and ^{G}neutrophils. (b) Upregulated genes: one gene (Actb) is commonly upregulated. (c) Downregulated genes: two genes (Cybb, Itgam) are commonly downregulated in both cell types.

Pathway enrichment was assessed using FDR correction. Numbers in Venn diagrams refer to gene counts.

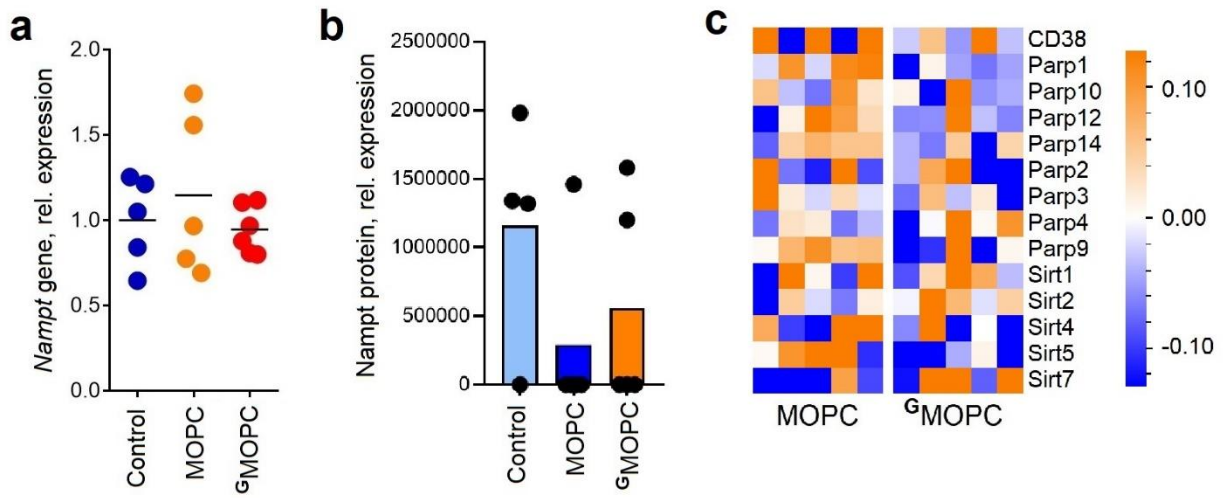

Figure S18 | Regulation of Nampt gene and protein expression in lung neutrophils from control, MOPC- and ^{G}MOPC-bearing mice.

(a) Relative mRNA expression of *Nampt* in lung neutrophils, determined by quantitative RT-PCR. Each dot represents an individual animal from control (n=5), MOPC (n=5), and ^{G}MOPC (n=6) groups; horizontal bars indicate group means.
(b) Quantification of Nampt protein expression in lung neutrophils from control (n=4), MOPC (n=5), and ^{G}MOPC (n=5) groups by proteomics. Bar graphs represent mean relative protein expression for each group, with individual values shown as black dots.
(c) Heatmap visualization of the abundance levels of key Nampt-consuming enzymes in lung tissue, assessed by quantitative proteomics in MOPC, and ^{G}MOPC models (n=5 mice in each group).
Statistical differences were assessed using Mann-Whitney test.

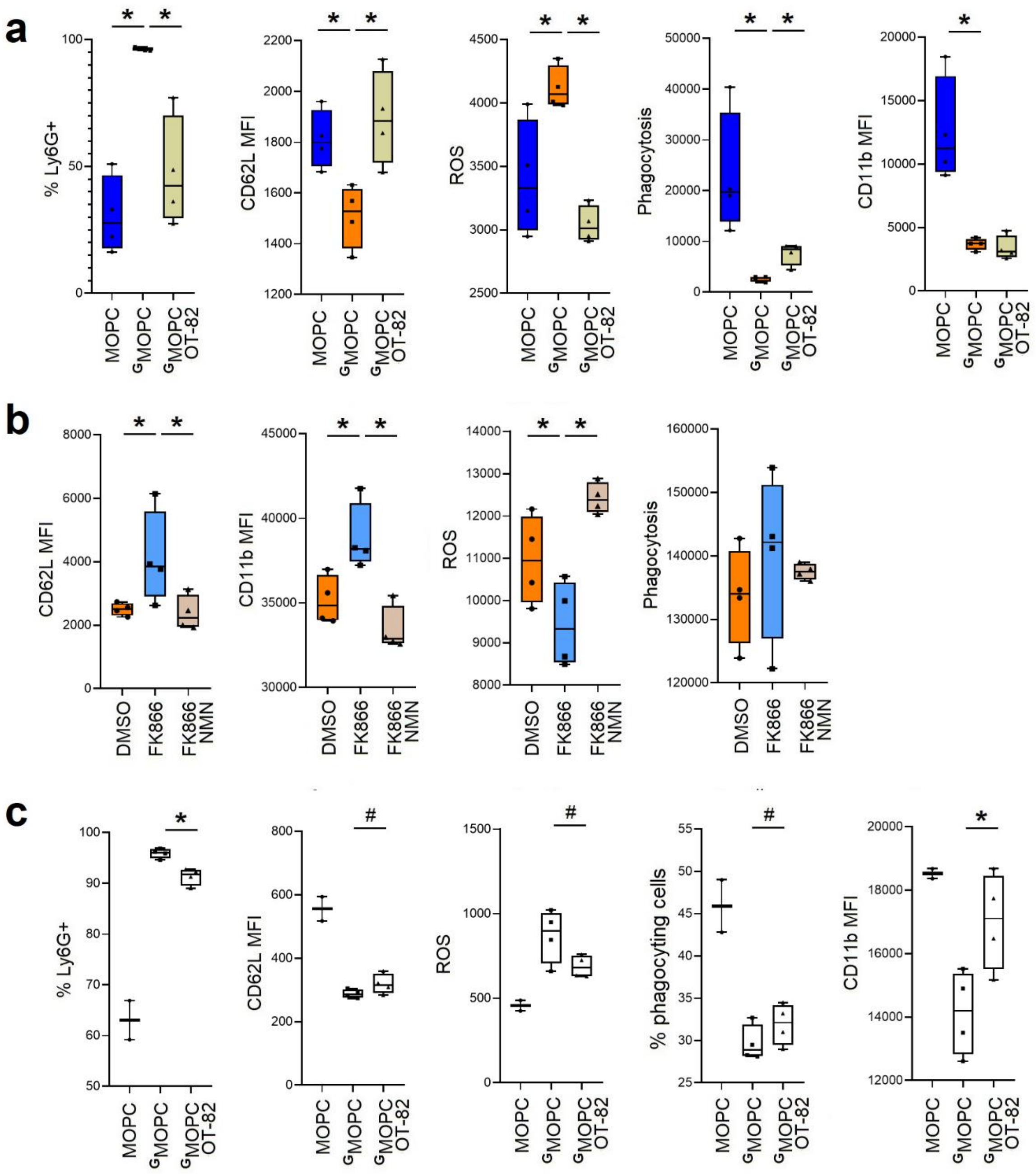

Figure S19 | Regulation of neutrophil maturation by NAMPT in tumor-conditioned environments *in vitro* and *in vivo*.

(a) *In vitro* maturation of isolated bone marrow progenitors isolated from n=4 tumor-naïve mice cultured in tumor-conditioned medium from MOPC or ^{G}MOPC tumor cell lines, with or without the NAMPT inhibitor OT82. Shown are quantifications of maturation markers (% Ly6G+ cells), CD62L median fluorescence intensity (MFI), reactive oxygen species (ROS) levels, phagocytic capacity, and CD11b MFI after 6 days of differentiation. Box-and-whisker plots represent interquartile ranges and medians; each box represents pooled independent experiments. *P < 0.05, #P < 0.1, Mann-Whitney test.

(b) *In vitro* maturation of bone marrow progenitors isolated from n=4 tumor-naïve mice in ^{G}MOPC-conditioned medium, with either the NAMPT inhibitor FK866 or co-administration of NAMPT metabolite NMN (nicotinamide mononucleotide). Shown are effects on CD62L and CD11b MFI, ROS, and phagocytic activity. Box-and-whisker plots represent interquartile ranges and medians. *P < 0.05, #P < 0.1, Mann-Whitney test.

(c) *In vivo* assessment of lung neutrophils in mice bearing MOPC (n=2), or ^{G}MOPC tumors, with (n=4 mice) or without (n=4 mice) OT82 treatment. Quantification includes percentage of Ly6G+ neutrophils, CD62L and CD11b MFI, ROS, and percentage of phagocytosing neutrophils in lung tissue. Box plots display median and interquartile ranges. *p < 0.05, #p < 0.1, Mann-Whitney test.

Supplementary table 1. Score sheet.

Score Sheet

Observation classification	points
**I Body weight**	
- unaffected or increase / BCS 3 (normal weight)	0
- Change <5%	1
- Weight reduction 5-10%	5
- Weight reduction 11-20% / BCS 2 (underweight)	10
- Weight reduction ≥ 20% / BCS 1 (cachexia)	20
**II General condition**	
- fur smooth, shiny; body openings clean; eyes clear, shiny	0
- Skin defects (reduced or excessive body care)	1
- Fur blunt, disorderly, unkempt body openings, eyes cloudy; increased muscle tone	5
- Dirty fur, sticky or damp body openings, abnormal attitude, eyes cloudy; high muscle tone	10
- cramps, paralysis (trunk muscles, extremities); wheezing; animal feels cold	20
**III Spontaneous behavior**	
- normal behavior (sleeping, reaction to blowing and touching, curiosity, social contacts)	0
- small deviations from the normal behavior	1
- abnormal behavior, impaired motor function or hyperkinetics	5
- self-isolation, lethargy; pronounced hyperkinetics or behavioral stereotypes, incoordination	10
- apathy; pain sounds when grasping; self-amputation (autoaggression)	20
**IV Clinical findings**	
- normal breathing, extremities warm	0
- small deviations from the normal situation	1
- respiratory rate noticeably increased	10
- dyspnea; tumor diameter at s.c. model > 1cm, ulcerative tumor	20

Rating, action	point total
Burden level 0 = no burden	0
Burden level 1 = low burden, carefully continue monitoring	1 - 9
Burden level 2 = moderate burden; control interval 2x daily; if necessary, initiate veterinary care; By lasting > 2 days classify as burden level 3	10 - 19
Burden level 3 = high burden; immediate termination of the experiment; kill animal painlessly	20 and higher

Supplementary table 2. Reagents and resources used in the study.

REAGENT or RESOURSE	SOURCE	IDENTIFIER (Cat#)
**Antibodies**		
Human Fc block	BD Biosciences	564220
CD16/CD32 (Mouse BD Fc Block), anti-Mouse, 0.5mg	BD Biosciences	553142
Pacific Blue™ anti-mouse/human CD11b Antibody	BioLegend	101224
Biotin anti-mouse/human CD11b Antibody clone M1/70 500 µg	BioLegend	101204
Biotin anti-mouse Ly-6G Antibody, 50ug	BioLegend	127603
Biotin anti-mouse CD19 Antibody, 50ug	BioLegend	115503
Biotin anti-mouse NK-1.1 Antibody, 50ug	BioLegend	108703
Rat anti-mouse Ly6G	BioLegend	127608
Anti mouse CD62L PE-Cyanine 7, 100ug	BioLegend	104418
FITC anti-mouse CD182 (CXCR2) Antibody, rt-IgG2a, 100µg	BioLegend	149310
CD184 (CXCR4) Monoclonal Antibody (2B11), Alexa Fluor™ 488, Rat / IgG2b, kappa, 25ug	eBioscience	53-9991-80
Pacific Blue anti-mouse H-2Kd Antibody, MHC class I, 100µg	BioLegend	116616
CD101 Monoclonal Antibody (Moushi101), PE, 25ug	eBioscience	12-1011-82
CSF3R Monoclonal Antibody (723806), anti-mouse, 100µg	ThermoFisher	MA5-24339
CD66b antihuman APC-Alexa Fluor 750, 0,5ml	Beckman Coulter	B08756
anti-human CD62L Pac blue 100 ug	BioLegend	304826
Anti-DNA/Histone H1 Antibody, 100µg	Merk Millipore	MAB3864
**Biological samples**		
Human head and neck squamous cell carcinoma tissues	University Hospital Essen	N/A
Human head and neck squamous cell carcinoma blood	University Hospital Essen	N/A
Human head and neck squamous cell carcinoma oral rinse	University Hospital Essen	N/A
**Chemicals, peptides, and recombinant proteins**		
DMEM Medium	Gibco	41966-029
RPMI 1640 Medium	Gibco	11875093
Fetal Bovine Serum Supreme (FBS)	PAN Biotech	P30-3031
Penicillin/Streptomycin 10.000 U/ml	Gibco	15140122
Sodium Pyruvate 100 mM	Gibco	11360070
3.2% sodium citrate s-Monovette	Sarstedt	21.067.001
Hams F-12 Nutrient Mix	Gibco	21765029
Hydrocortisone	Sigma-Aldrich/ Merck	H0888
Cholera Toxin	Sigma-Aldrich/ Merck	C8052
Transferrin	Sigma-Aldrich/ Merck	T3309
Insulin	Sigma-Aldrich/ Merck	I6634
Triiodo-L-thyronine	Sigma-Aldrich/ Merck	T6397
Epidermal Growth Factor (E.G.F.)	Sigma-Aldrich/ Merck	E4127
Dulbecco's Phosphate Buffered Saline 1x PBS	Gibco	14040133
Collagenase	Roche / Merck	11088866001

Deoxyribonuclease I	Merck	DN25-100MG
Dispase II	Sigma-Aldrich/ Merck	D4693-1G
Biocoll Separation Solution (density 1,077 g/ml)	Merck	L6115
eBioscience Fixable Viability Dye	Thermo Fisher Scientific	65-0865-14, 65-0866-14
PE Annexin V Apoptosis Detection Kit I	BD Biosciences	559763
RNAlater RNA stabilizing solution	Invitrogen	AM7020
Anti-Biotin MicroBeads	Miltenyi Biotec	130-090-485
cOmplete™, Mini, EDTA-freier Protease-Inhibitor-Cocktail	Merck Roche	4693159001
mrSCF	Peprotech	
mrIL3	Peprotech	
PMA: Phorbol 12-myristate 13-acetate	Biomol	Cay-10008014-1
Lipopolysaccharid aus Pseudomonas aeruginosa 10	Sigma-Aldrich	L9143-10MG
FK866 hydrochloride	Axon Medchem	Axon 1546
SYTOX Green Dead Cell Stain	Invitrogen	S34860
Dihydrorhodamine 123	Sigma-Aldrich	D1054-2MG
CFSE Cell Division Tracker Kit	BioLegend	423801
Phagocytosis Assay kit	Cayman Chemical	500290
PKH26 Red Fluorescent Cell Linker Midi Kit	Sigma Aldrich	MIDI26-1KT
PureBlu Hoechst 33342 Nuclear staining Dye	BIoRad	1351304
DAPI (4',6-Diamidino-2-Phenylindole, Dilactate)	BioLegend	422801
QVD-OPh, caspase inhibitor	abcam	ab141421
Paraformaldehyde 16% w/v aq.soln., methanol free liquid, ampouled under argon, 10x10 ml	Alfa Aesar	43368
ProLong Gold Antifade Mountant with DAPI	Invitrogen	P36935
Poly-D-lysine hydrobromide	Sigma-Aldrich	P0899-10MG
**Critical commercial assays**		
Human G-CSF ELISA	R&D Systems	
Mouse G-CSF DuoSet ELISA	R&D Systems	DY414-05
Mouse TNF-alpha DuoSet ELISA, 5 Plates	R&D Systems	DY410-05
NAD/NADH Assay kit (Colorimetric)	Abcam	ab65348
Qia Shredder 250	Qiagen	79656
RNeasy Mini 250 Kit	Qiagen	74106
SuperScript II Reverse Transcriptase	Invitrogen	18064014
Luna Universal qPCR Master Mix	BioLabs	M3003X
**Deposited data**		
Proteome data are deposited as PXD052631 (https://www.ebi.ac.uk/pride/archive/projects/PXD052631) and PXD069569 (https://www.ebi.ac.uk/pride/archive/projects/PXD069569) databases	RroteomeXChange, PRIDE database	PXD052631, PXD069569
Microarray data deposited in the Gene Expression Omnibus database blood cells	https://ncbi.nlm.nih.gov/geo/query/acc.cgi?acc=GSE11247	GEO: GSE11247
**Experimental models: cell lines**		
MOPC	Dr. William Chad Spanos and John H. Lee (Sanford Research/ University of South Dakota)	N/A

^{G}MOPC	Dr. H. Hananbers (originally from Dr. William Chad Spanos and John H. Lee (Sanford Research/ University of South Dakota))	N/A
**Experiments models: Organisms/strains**		
Mouse: C57BL/6JCrl	Dr. Jadwiga Jablonska (University Hospital Essen), originally JAX strain from Charles River Laboratory	RRID:MGI: 3775640
Pseudomonas aeruginosa PA14		
**Oligonucleotides**		
PCR-forward for Mpo: 5‘-CGTGTCAAGTGGCTGTGCCTAT-3‘	Thermo Fisher Scientific	N/A
PCR-reverse for Mpo: 5‘-AACCAGCGTACAAAGGCACGGT-3‘	Thermo Fisher Scientific	N/A
PCR-forward for Mmp9: 5‘-GCTGACTACGATAAGGACGGCA-3‘	Thermo Fisher Scientific	N/A
PCR-reverse for Mmp9: 5‘-TAGTGGTGCAGGCAGAGTAGGA-3‘	Thermo Fisher Scientific	N/A
PCR-forward for Rps9: 5‘-TTGACGCTAGACGAGAAGGAT-3‘	Thermo Fisher Scientific	N/A
PCR-reverse for Rps9: 5‘-AATCCAGCTTCATCTTGCCCT-3‘	Thermo Fisher Scientific	N/A
PCR-forward for Caspase3: 5‘-ATGGGAGCAAGTCAGTGGAC-3‘	Thermo Fisher Scientific	N/A
PCR-reverse for Caspase3: 5‘-TTGAGGTAGCTGCACTGTGG-3‘	Thermo Fisher Scientific	N/A
PCR-forward for Nampt: 5'-TAC AGTGGCCACAAATTCCA-3'	Thermo Fisher Scientific	N/A
PCR-reverse for Nampt: 5'-CAATTCCCGCCA CAGTATCT-3'	Thermo Fisher Scientific	N/A
**Consumables**		
LD Columns, 25 Stück	Miltenyi Biotec	130-042-901
pluriStrainer 50 µm (Cell Strainer), 50 Pack, unsterile	PluriSelect	43-50050-03
pluriStrainer 100 µm (Cell Strainer), 50 Pack, unsterile	PluriSelect	43-50100-51
96-Well-Platte, No. 1.5 Coverslip, 5mm Glass Diameter, uncoated, 5 Platten	MatTec	P96G-1.5-5-F
**Software and algorithms**		
BD FACS Diva v8	BD Biosciences	N/A
ImageJ FIJI	Rasband, W.S.	https://imagej.nih.gov/ij/, 1997-2018
GraphPad Prism	GraphPad software	
Qlucore Omics explorer 3.10	Qlucore	
open access ShinyGO platform v. 0.78	Ge SX, Jung D & Yao R, Bioinformatics 36:2628–2629, 2020	http://bioinformatics.sdstate.edu/go/
MedCalc’s calculators		https://www.medcalc.org/calc/
