# Supplementary material for: G-CSF/NAMPT signaling drives neutrophil dysfunction and enhances bacterial infection susceptibility in cancer patients
Source: Nat Commun. 2025 Dec 12;16:11137. doi: 10.1038/s41467-025-67471-4 (PMC12705696; doi:10.1038/s41467-025-67471-4)
Supplement: Supplementary file 2 — Description of Additional Supplementary Files [file 41467_2025_67471_MOESM2_ESM.pdf]

Description of Additional Supplementary Files:

Supplementary Data 1: "List of differentially expressed proteins from neutrophil proteomic analysis described in Figure 2, with associated fold-changes and statistical values."

Supplementary Data 2: "GO enrichment analysis dataset supporting Figure 2j–l, listing the biological processes downregulated in lung neutrophils upon chronic G-CSF exposure."

Supplementary Data 3: "GO enrichment analysis dataset supporting Figure 2j–l, listing the biological processes upregulated in lung neutrophils upon chronic G-CSF exposure."

Supplementary Data 4: "GO enrichment analysis dataset supporting Figure 2j–l, listing the cellular compartments downregulated in lung neutrophils upon chronic G-CSF exposure."

Supplementary Data 5: "GO enrichment analysis dataset supporting Figure 2j–l, listing the cellular compartments upregulated in lung neutrophils upon chronic G-CSF exposure."

Supplementary Data 6: "GO enrichment analysis dataset supporting Figure 2j–l, listing the molecular functions downregulated in lung neutrophils upon chronic G-CSF exposure."

Supplementary Data 7: "GO enrichment analysis dataset supporting Figure 2j–l, listing the molecular functions upregulated in lung neutrophils upon chronic G-CSF exposure."
