# Supplementary material for: G-CSF/NAMPT signaling drives neutrophil dysfunction and enhances bacterial infection susceptibility in cancer patients
Source: Nat Commun. 2025 Dec 12;16:11137. doi: 10.1038/s41467-025-67471-4 (PMC12705696; doi:10.1038/s41467-025-67471-4)
Supplement: Supplementary file 10 — Reporting Summary [file 41467_2025_67471_MOESM10_ESM.pdf]

nature portfolio

Corresponding author(s): Jadwiga Jablonska

Last updated by author(s): 27.10.2025

Reporting Summary

Nature Portfolio wishes to improve the reproducibility of the work that we publish. This form provides structure for consistency and transparency in reporting. For further information on Nature Portfolio policies, see our Editorial Policies and the Editorial Policy Checklist.

Statistics

For all statistical analyses, confirm that the following items are present in the figure legend, table legend, main text, or Methods section.

n/a	Confirmed	
☐	☒	The exact sample size (*n*) for each experimental group/condition, given as a discrete number and unit of measurement
☐	☒	A statement on whether measurements were taken from distinct samples or whether the same sample was measured repeatedly
☐	☒	The statistical test(s) used AND whether they are one- or two-sided<br>*Only common tests should be described solely by name; describe more complex techniques in the Methods section.*
☐	☒	A description of all covariates tested
☐	☒	A description of any assumptions or corrections, such as tests of normality and adjustment for multiple comparisons
☐	☒	A full description of the statistical parameters including central tendency (e.g. means) or other basic estimates (e.g. regression coefficient) AND variation (e.g. standard deviation) or associated estimates of uncertainty (e.g. confidence intervals)
☐	☒	For null hypothesis testing, the test statistic (e.g. *F*, *t*, *r*) with confidence intervals, effect sizes, degrees of freedom and *P* value noted<br>*Give P values as exact values whenever suitable.*
☒	☐	For Bayesian analysis, information on the choice of priors and Markov chain Monte Carlo settings
☒	☐	For hierarchical and complex designs, identification of the appropriate level for tests and full reporting of outcomes
☐	☒	Estimates of effect sizes (e.g. Cohen's *d*, Pearson's *r*), indicating how they were calculated

Our web collection on statistics for biologists contains articles on many of the points above.

Software and code

Policy information about availability of computer code

Data collection	Data acquisition was performed using the following software platforms: ZEN Blue 2012 (Carl Zeiss), CellSens Dimension (Olympus), BD FACS Diva Software 9.0 (BD Biosciences), and Gen5 3.09 (BioTek). These programs were used for imaging, flow cytometry, and plate reader data processing as appropriate for each experimental modality.
Data analysis	LC-MS raw data were processed using the directDIA algorithm in Spectronaut (Version 19.1.240806.626, Biognosys, Schlieren, Switzerland) with BSG factory settings (Enzyme/Cleavage rules: Trypsin/P; Fixed modification: Carbamidomethyl (C); Variable modifications: Acetyl (Protein N-term), Oxidation (M)). Data were searched against a reviewed mouse UniProt/Swissprot FASTA database (downloaded on 13th March 2024; 17,196 target sequences). LC-MS/MS data from DDA experiments were analyzed using the Sequest algorithm within Proteome Discoverer (v2.4.1.15, Thermo Fisher Scientific). Flow cytometry data were analyzed with FlowJo v10.10.0, pathway analyses were performed using ShinyGO (Version 0.78), and further data exploration was done with Qlucore Omics Explorer v3.10. Statistical analysis and graphing were performed using Prism v8.0.

For manuscripts utilizing custom algorithms or software that are central to the research but not yet described in published literature, software must be made available to editors and reviewers. We strongly encourage code deposition in a community repository (e.g. GitHub). See the Nature Portfolio guidelines for submitting code & software for further information.

Data

Policy information about availability of data

All manuscripts must include a data availability statement. This statement should provide the following information, where applicable:

- Accession codes, unique identifiers, or web links for publicly available datasets
- A description of any restrictions on data availability
- For clinical datasets or third party data, please ensure that the statement adheres to our policy

The mass spectrometry proteomics data have been deposited to the ProteomeXchange Consortium via the PRIDE partner repository with the dataset identifier PXD052631 and PXD069569. Source data are provided with the paper.

Research involving human participants, their data, or biological material

Policy information about studies with human participants or human data. See also policy information about sex, gender (identity/presentation), and sexual orientation and race, ethnicity and racism.

Reporting on sex and gender	Sex of participants was recorded and included in the study analyses. Both male and female participants were enrolled from patient and healthy control groups. Gender identity was not assessed, as it was not relevant to the scientific objectives of this study. The influence of biological sex on the frequency of pathogen detection was specifically analyzed, given established evidence of sex-based differences in infection predisposition and immune response. The results from these analyses are presented in the supplementary data and main tables where relevant.
Reporting on race, ethnicity, or other socially relevant groupings	Race, ethnicity, and other socially relevant groupings were not assessed in this study. These variables were not relevant to the scientific objectives or context of the research and therefore were not included in participant recruitment or data analysis.
Population characteristics	Relevant population characteristics were recorded for all participants, including sex, age, smoking status (categorized as never, past, or current), and HPV status. These variables were selected due to their established importance and relevance to disease outcomes in this study. Analyses related to these characteristics are presented in the Results and Supplementary sections.
Recruitment	Patients receiving treatment in the ENT department of University Hospital Essen were recruited for the study. Healthy volunteers were also enrolled. No recruitment biases were identified, and all participants provided informed consent before participation.
Ethics oversight	The ethics committee of the University Hospital Essen, Germany (19-8599-BO, 16-7135-BO)

Note that full information on the approval of the study protocol must also be provided in the manuscript.

Field-specific reporting

Please select the one below that is the best fit for your research. If you are not sure, read the appropriate sections before making your selection.

[x] Life sciences [ ] Behavioural & social sciences [ ] Ecological, evolutionary & environmental sciences

For a reference copy of the document with all sections, see nature.com/documents/nr-reporting-summary-flat.pdf

Life sciences study design

All studies must disclose on these points even when the disclosure is negative.

Sample size	For patient and healthy volunteer cohorts, all available eligible material and participants at the time of the study were included, without predetermined sample size calculation. This approach aimed to maximize the use of accessible material and enhance data representativeness for the cohort. For animal and in vitro experiments, sample size was based on field standards and practical feasibility, with 5–6 animals per group.
Data exclusions	No data were excluded
Replication	All experiments were performed in biological replicates. Experiments were independently repeated 2–3 times to ensure reproducibility. Experimental group sizes, total numbers, and replication details are provided in figure legends and Methods.
Randomization	The experiments with human material were not randomized. In murine experiments, mice were randomized into the groups.
Blinding	The investigators were blinded during data analysis

Reporting for specific materials, systems and methods

We require information from authors about some types of materials, experimental systems and methods used in many studies. Here, indicate whether each material, system or method listed is relevant to your study. If you are not sure if a list item applies to your research, read the appropriate section before selecting a response.

Materials & experimental systems

n/a	Involved in the study
☐	☒ Antibodies
☐	☒ Eukaryotic cell lines
☐	☐ Palaeontology and archaeology
☐	☒ Animals and other organisms
☐	☒ Clinical data
☐	☐ Dual use research of concern
☐	☐ Plants

Methods

n/a	Involved in the study
☐	☐ ChIP-seq
☐	☒ Flow cytometry
☐	☐ MRI-based neuroimaging

Antibodies

Antibodies used

The following antibodies were utilized for flow cytometry and immunostaining:
Human Fc block (BD Biosciences, 564220)
Mouse BD Fc Block, CD16/CD32 (BD Biosciences, 553142)
Anti-mouse/human CD11b, Pacific Blue™ conjugate (BioLegend, 101224)
Anti-mouse/human CD11b, Biotin clone M1/70 (BioLegend, 101204)
Anti-mouse Ly-6G, Biotin conjugate (BioLegend, 127603)
Anti-mouse CD19, Biotin conjugate (BioLegend, 115503)
Anti-mouse NK-1.1, Biotin conjugate (BioLegend, 108703)
Rat anti-mouse Ly6G (BioLegend, 127608)
Anti-mouse CD62L, PE-Cyanine 7 conjugate (BioLegend, 104418)
Anti-mouse CD182 (CXCR2), FITC conjugate (BioLegend, 149310)
Anti-mouse CD184 (CXCR4), Alexa Fluor™ 488 conjugate (clone 2B11, eBioscience, 53-9991-80)
Anti-mouse H-2Kd (MHC class I), Pacific Blue conjugate (BioLegend, 116616)
Anti-mouse CD101, PE conjugate (clone Moushi101, eBioscience, 12-1011-82)
Anti-mouse CSF3R (clone 723806, ThermoFisher, MA5-24339)
Anti-human CD66b, APC-Alexa Fluor 750 conjugate (Beckman Coulter, B08756)
Anti-human CD62L, Pacific Blue conjugate (BioLegend, 304826)
Anti-DNA/Histone H1 antibody (Merk Millipore, MAB3864)
Antibodies were sourced from BD Biosciences, BioLegend, eBioscience, ThermoFisher, Beckman Coulter, and Merk Millipore, with relevant clones and fluorochrome conjugates indicated.

Validation

All antibodies used in this study were validated by the respective manufacturers. Validation protocols, specificity data, and quality controls are provided by the suppliers and referenced in their product documentation or datasheets.

Eukaryotic cell lines

Policy information about cell lines and Sex and Gender in Research

Cell line source(s)

The murine oropharyngeal carcinoma cell line MOPC (C57BL/6-derived, HPV16 E6/E7 negative) was obtained from Dr. William Chad Spanos and John H. Lee (Sanford Research/University of South Dakota, USA). Modified cell lines expressing low and high G-CSF were generated via CRISPR/Cas9-mediated knock-in technology.

Authentication

Cell line identity was verified using CRISPR/Cas9 targeted knock-in approaches and regular phenotypic validation. Molecular phenotype and function were carefully assessed throughout experiments to confirm authentication

Mycoplasma contamination

All cell lines were routinely tested for mycoplasma contamination throughout cultivation, with consistently negative results

Commonly misidentified lines
(See ICLAC register)

None of the cell lines used in this study are listed on the ICLAC register of misidentified or contaminated cell lines

Palaeontology and Archaeology

Specimen provenance

Provide provenance information for specimens and describe permits that were obtained for the work (including the name of the issuing authority, the date of issue, and any identifying information). Permits should encompass collection and, where applicable, export.

Specimen deposition

Indicate where the specimens have been deposited to permit free access by other researchers.

Dating methods

If new dates are provided, describe how they were obtained (e.g. collection, storage, sample pretreatment and measurement), where

they were obtained (i.e. lab name), the calibration program and the protocol for quality assurance OR state that no new dates are provided.

☐ Tick this box to confirm that the raw and calibrated dates are available in the paper or in Supplementary Information.

Ethics oversight	*Identify the organization(s) that approved or provided guidance on the study protocol, OR state that no ethical approval or guidance was required and explain why not.*

Note that full information on the approval of the study protocol must also be provided in the manuscript.

Animals and other research organisms

Policy information about studies involving animals; ARRIVE guidelines recommended for reporting animal research, and Sex and Gender in Research

Laboratory animals	Female and male C57BL/6JCrl mice (8–12 weeks old) were used, bred and housed under specific pathogen-free conditions at University Hospital Essen. All animal experiments were conducted according to German law and FELASA recommendations
Wild animals	No wild animals were included in this study; only laboratory-bred C57BL/6 mice were used
Reporting on sex	Sex of both human and animal participants was recorded and considered in analyses. Both male and female humans (patients and healthy controls) and male and female mice were included. The influence of sex on study outcomes, such as pathogen detection rates, was analyzed
Field-collected samples	No field-collected samples were used. All biological samples (human, animal, cell lines) were obtained under controlled laboratory or clinical conditions
Ethics oversight	Human experiments were approved by the ethics committee of the University Hospital Essen, Germany (19-8599-BO, 16-7135-BO). Animal experiments were approved by LANUV (Das Landesamt für Natur, Umwelt und Verbraucherschutz Nordrhein-Westfalen, Germany) and performed in accordance with national and FELASA guidelines.

Note that full information on the approval of the study protocol must also be provided in the manuscript.

Clinical data

Policy information about clinical studies

All manuscripts should comply with the ICMJE guidelines for publication of clinical research and a completed CONSORT checklist must be included with all submissions.

Clinical trial registration	This study does not report clinical trial registration, as it was not a clinical trial.
Study protocol	Full experimental protocols for both animal and human studies are detailed within the materials and methods sections of the manuscript, and adhere to the relevant regulatory guidelines for preclinical and observational research.
Data collection	Data were collected using standardized laboratory procedures for cell culture, animal handling, sample acquisition (including oral rinse, blood, tissue, and bacterial cultures), as well as validated imaging, flow cytometry, proteomics, and statistical software
Outcomes	Primary study outcomes comprised evaluation of neutrophil dysfunction and bacterial infection susceptibility in cancer patients and animal models, including cellular, molecular, and clinical endpoints detailed in the Results and Supplementary Information

Dual use research of concern

Policy information about dual use research of concern

Hazards

Could the accidental, deliberate or reckless misuse of agents or technologies generated in the work, or the application of information presented in the manuscript, pose a threat to:

No	Yes	
☒	☐	Public health
☒	☐	National security
☒	☐	Crops and/or livestock
☒	☐	Ecosystems
☒	☐	Any other significant area

Experiments of concern

Does the work involve any of these experiments of concern:

No	Yes	
☒	☐	Demonstrate how to render a vaccine ineffective
☒	☐	Confer resistance to therapeutically useful antibiotics or antiviral agents
☒	☐	Enhance the virulence of a pathogen or render a nonpathogen virulent
☒	☐	Increase transmissibility of a pathogen
☒	☐	Alter the host range of a pathogen
☒	☐	Enable evasion of diagnostic/detection modalities
☒	☐	Enable the weaponization of a biological agent or toxin
☒	☐	Any other potentially harmful combination of experiments and agents

Plants

Seed stocks

Report on the source of all seed stocks or other plant material used. If applicable, state the seed stock centre and catalogue number. If plant specimens were collected from the field, describe the collection location, date and sampling procedures.

Novel plant genotypes

Describe the methods by which all novel plant genotypes were produced. This includes those generated by transgenic approaches, gene editing, chemical/radiation-based mutagenesis and hybridization. For transgenic lines, describe the transformation method, the number of independent lines analyzed and the generation upon which experiments were performed. For gene-edited lines, describe the editor used, the endogenous sequence targeted for editing, the targeting guide RNA sequence (if applicable) and how the editor was applied.

Authentication

Describe any authentication procedures for each seed stock used or novel genotype generated. Describe any experiments used to assess the effect of a mutation and, where applicable, how potential secondary effects (e.g. second site T-DNA insertions, mosiacism, off-target gene editing) were examined.

ChIP-seq

Data deposition

☐ Confirm that both raw and final processed data have been deposited in a public database such as GEO.

☐ Confirm that you have deposited or provided access to graph files (e.g. BED files) for the called peaks.

Data access links
May remain private before publication.

For "Initial submission" or "Revised version" documents, provide reviewer access links. For your "Final submission" document, provide a link to the deposited data.

Files in database submission

Provide a list of all files available in the database submission.

Genome browser session
(e.g. UCSC)

Provide a link to an anonymized genome browser session for "Initial submission" and "Revised version" documents only, to enable peer review. Write "no longer applicable" for "Final submission" documents.

Methodology

Replicates

Describe the experimental replicates, specifying number, type and replicate agreement.

Sequencing depth

Describe the sequencing depth for each experiment, providing the total number of reads, uniquely mapped reads, length of reads and whether they were paired- or single-end.

Antibodies

Describe the antibodies used for the ChIP-seq experiments; as applicable, provide supplier name, catalog number, clone name, and lot number.

Peak calling parameters

Specify the command line program and parameters used for read mapping and peak calling, including the ChIP, control and index files used.

Data quality

Describe the methods used to ensure data quality in full detail, including how many peaks are at FDR 5% and above 5-fold enrichment.

Software

Describe the software used to collect and analyze the ChIP-seq data. For custom code that has been deposited into a community repository, provide accession details.

Flow Cytometry

Plots

Confirm that:

- [x] The axis labels state the marker and fluorochrome used (e.g. CD4-FITC).
- [x] The axis scales are clearly visible. Include numbers along axes only for bottom left plot of group (a 'group' is an analysis of identical markers).
- [x] All plots are contour plots with outliers or pseudocolor plots.
- [x] A numerical value for number of cells or percentage (with statistics) is provided.

Methodology

Sample preparation	Single-cell suspensions were prepared from murine (lung, bone marrow, blood, tumor) and human (blood, oral rinse, or tumor) tissues using enzymatic digestion and filtration where appropriate. Red blood cells were lysed (ACK buffer). Samples were stained with validated panels of fluorochrome-conjugated antibodies targeting surface markers. For functional assays, viable neutrophils or neutrophil subsets were isolated by flow cytometric sorting (FACS) or used for downstream in vitro functional analysis. Details of staining, washing, and fixation (where needed) are provided in the Methods section.
Instrument	All flow cytometry measurements were performed using BD FACS Aria (for cell sorting), BD FACS Canto II, and BD LSRFortessa instruments (BD Biosciences, USA).
Software	Data acquisition was performed with BD FACS Diva Software v9.0 (BD Biosciences), and analysis was conducted using FlowJo v10.10.0 (BD Biosciences). Additional analysis and visualization were conducted with GraphPad Prism 8.0 and Qlucore Omics Explorer 3.10 as appropriate for statistical evaluation.
Cell population abundance	Populations of interest (e.g., total neutrophils, Ly6G^+ cells, CD11b^+, CD62L^low^, or CD66b^+^ human neutrophils) were quantified as percentages and absolute numbers among viable leukocytes or total single cells. Detailed results for abundance and subset frequencies are reported in the main text, figures, and supplementary data.
Gating strategy	Gating strategies for murine and human flow cytometry are detailed in the Supplementary Figures (e.g., Supplementary Figure S3). Live single cells were first selected based on forward/side scatter, exclusion of doublets, and viability dye. Leukocytes were gated as CD45^+^, and neutrophils as Ly6G^+^CD11b^+^ in mice or CD66b^+^ in humans. Subsets (e.g., CD62L^low^, CD101^high^, CXCR4^high^) were then identified by further sequential gating. All gates and representative plots are shown in the respective figures and described in the Methods section

- [x] Tick this box to confirm that a figure exemplifying the gating strategy is provided in the Supplementary Information.

Magnetic resonance imaging

Experimental design

Design type	*Indicate task or resting state; event-related or block design.*
Design specifications	*Specify the number of blocks, trials or experimental units per session and/or subject, and specify the length of each trial or block (if trials are blocked) and interval between trials.*
Behavioral performance measures	*State number and/or type of variables recorded (e.g. correct button press, response time) and what statistics were used to establish that the subjects were performing the task as expected (e.g. mean, range, and/or standard deviation across subjects).*

Acquisition

Imaging type(s)	*Specify: functional, structural, diffusion, perfusion.*
Field strength	*Specify in Tesla*
Sequence & imaging parameters	*Specify the pulse sequence type (gradient echo, spin echo, etc.), imaging type (EPI, spiral, etc.), field of view, matrix size, slice thickness, orientation and TE/TR/flip angle.*
Area of acquisition	*State whether a whole brain scan was used OR define the area of acquisition, describing how the region was determined.*

Diffusion MRI [ ] Used [ ] Not used

Preprocessing

Preprocessing software	*Provide detail on software version and revision number and on specific parameters (model/functions, brain extraction, segmentation, smoothing kernel size, etc.).*
Normalization	*If data were normalized/standardized, describe the approach(es): specify linear or non-linear and define image types used for*

transformation OR indicate that data were not normalized and explain rationale for lack of normalization.

Normalization template

Describe the template used for normalization/transformation, specifying subject space or group standardized space (e.g. original Talairach, MNI305, ICBM152) OR indicate that the data were not normalized.

Noise and artifact removal

Describe your procedure(s) for artifact and structured noise removal, specifying motion parameters, tissue signals and physiological signals (heart rate, respiration).

Volume censoring

Define your software and/or method and criteria for volume censoring, and state the extent of such censoring.

Statistical modeling & inference

Model type and settings

Specify type (mass univariate, multivariate, RSA, predictive, etc.) and describe essential details of the model at the first and second levels (e.g. fixed, random or mixed effects; drift or auto-correlation).

Effect(s) tested

Define precise effect in terms of the task or stimulus conditions instead of psychological concepts and indicate whether ANOVA or factorial designs were used.

Specify type of analysis: ☐ Whole brain ☐ ROI-based ☐ Both

Statistic type for inference

(See Eklund et al. 2016)

Specify voxel-wise or cluster-wise and report all relevant parameters for cluster-wise methods.

Correction

Describe the type of correction and how it is obtained for multiple comparisons (e.g. FWE, FDR, permutation or Monte Carlo).

Models & analysis

n/a	Involved in the study
☐	☐ Functional and/or effective connectivity
☐	☐ Graph analysis
☐	☐ Multivariate modeling or predictive analysis

Functional and/or effective connectivity

Report the measures of dependence used and the model details (e.g. Pearson correlation, partial correlation, mutual information).

Graph analysis

Report the dependent variable and connectivity measure, specifying weighted graph or binarized graph, subject- or group-level, and the global and/or node summaries used (e.g. clustering coefficient, efficiency, etc.).

Multivariate modeling and predictive analysis

Specify independent variables, features extraction and dimension reduction, model, training and evaluation metrics.
